# Cerebral small vessel disease phenotype and 5-year mortality in asymptomatic middle-to-old aged individuals

**DOI:** 10.1038/s41598-021-02656-7

**Published:** 2021-11-30

**Authors:** Wei-Ju Lee, Kun-Hsien Chou, Pei-Lin Lee, Li-Ning Peng, Pei-Ning Wang, Ching-Po Lin, Liang-Kung Chen, Chih-Ping Chung

**Affiliations:** 1 Department of Family Medicine, Taipei Veterans General Hospital Yuanshan Branch, Yi-Lan, Taiwan; 2 Aging and Health Research Center, National Yang Ming Chiao Tung University College of Medicine, Taipei, Taiwan; 3 Institute of Neuroscience, National Yang Ming Chiao Tung University College of Medicine, Taipei, 11221 Taiwan; 4 Brain Research Center, National Yang Ming Chiao Tung University College of Medicine, Taipei, Taiwan; 5 School of Medicine, National Yang Ming Chiao Tung University College of Medicine, Taipei, Taiwan; 6 Center for Geriatric and Gerontology, Taipei Veterans General Hospital, Taipei, Taiwan; 7 Department of Neurology, Neurological Institute, Taipei Veterans General Hospital, No. 201, Section 2, Shipai Road, Beitou District, Taipei City, 112 Taiwan; 8 Taipei Municipal Gan-Dau Hospital (Managed By Taipei Veterans General Hospital), Taipei, Taiwan

**Keywords:** Diseases, Neurology

## Abstract

The present study aimed to determine whether a recently proposed cerebral small vessel disease (CSVD) classification scheme could differentiate the 5-year all-cause mortality in middle-to-old aged asymptomatic CSVD. Stroke-free and non-demented participants recruited from the community-based I-Lan Longitudinal Aging Study underwent baseline brain magnetic resonance imaging (MRI) between 2011 and 2014 and were followed-up between 2018 and 2019. The study population was classified into control (non-CSVD) and CSVD type 1–4 groups based on MRI markers. We determined the association with mortality using Cox regression models, adjusting for the age, sex, and vascular risk factors. A total of 735 participants were included. During a mean follow-up of 5.7 years, 62 (8.4%) died. There were 335 CSVD type 1 (57.9 ± 5.9 years), 249 type 2 (65.6 ± 8.1 years), 52 type 3 (67.8 ± 9.2 years), and 38 type 4 (64.3 ± 9.0 years). Among the four CSVD types, CSVD type 4 individuals had significantly higher all-cause mortality (adjusted hazard ratio = 5.0, 95% confidence interval 1.6–15.3) compared to controls. This novel MRI-based CSVD classification scheme was able to identify individuals at risk of mortality at an asymptomatic, early stage of disease and might be applied for future community-based health research and policy.

## Introduction

CNS (central nervous system) or cerebral small vessel disease (CSVD) causes 25% of stroke^1^. It is age-related and also considered as an important etiology of geriatric syndromes such as dementia, gait disturbance, and mood disorders^1,2^. The etiologies of age-related CSVD are heterogeneous and include two most common forms, arteriosclerosis/lipohyalinosis and amyloid accumulation (cerebral amyloid angiopathy; CAA)^1–3^. These brain microvascular pathologies might result in brain parenchymal damage through ischemia, edema or hemorrhage, particularly in the cerebral white matter^1–3^. Owing to developments in magnetic resonance imaging (MRI), we can now identify the presence of CSVD by visualizing the associated brain parenchyma changes at pre-mortem^2,4^. MRI markers of SVD are also heterogeneous and include ischemic lesions, such as white matter hyperintensities (WMH) and lacune(s), and hemorrhagic lesions, the cerebral microbleeds (CMB)^2,5^. These CSVD-related brain abnormalities usually co-occur in different etiologies, and their clinical courses are variable^6^. Clinical significances of each MRI CSVD marker have been revealed in several studies, particular in patients with stroke or dementia^1,2,5,6^. However, since each individual with CSVD usually has different combinations and severity of MRI markers, the known clinical risks of each single MRI marker might be hard to be applied in predicting individual’s clinical outcomes.

**Figure 1:**
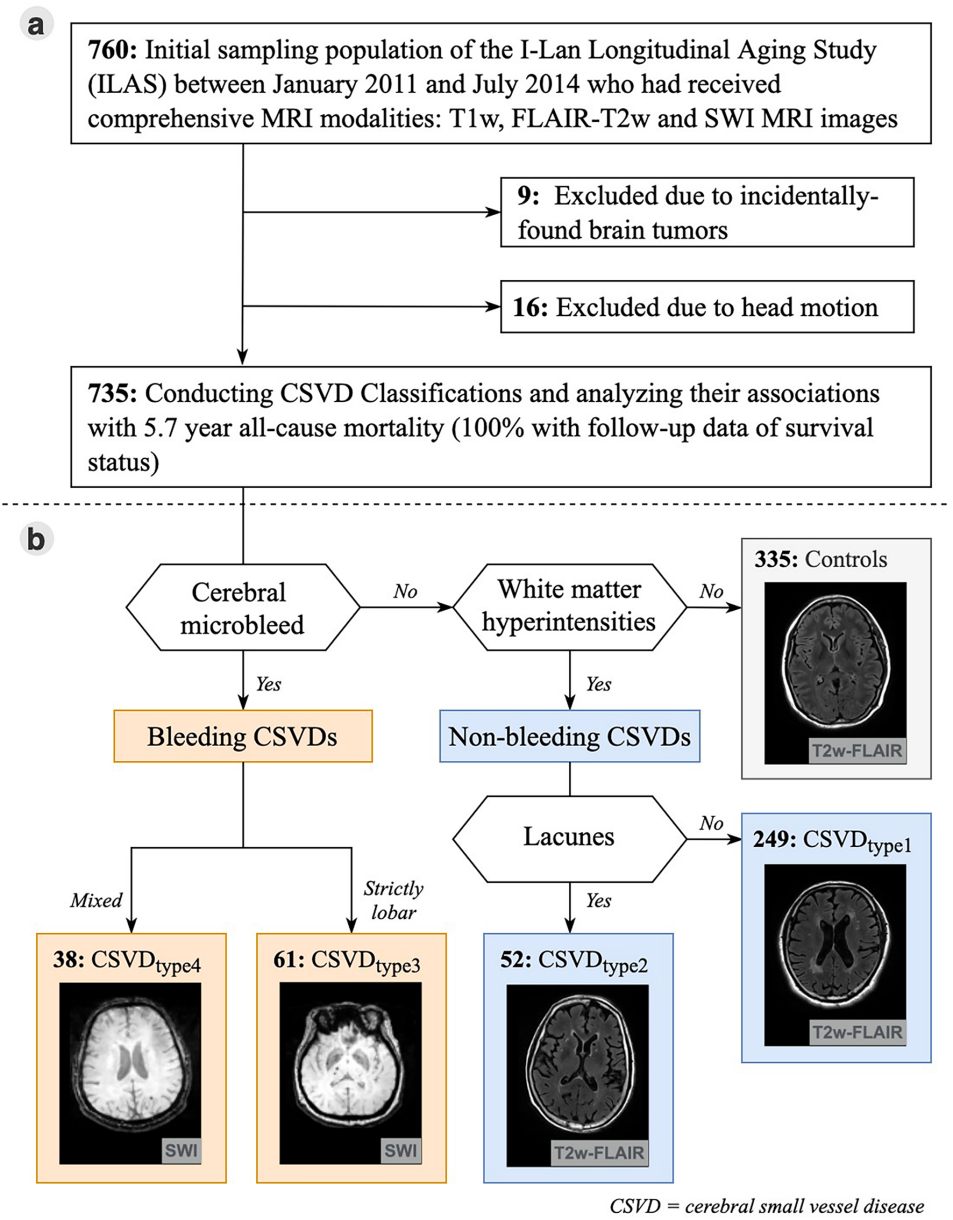
(**a**) Flowchart of included participants and analyzed neuroimaging data. (**b**) Methodological sequence for phenotyping age-related cerebral small vessel disease. The stratification scheme had three steps in the following order: (1) presence of cerebral microbleeds, (2) presence of severe WMH (defined as > 50th percentile of WMH/TIV ratio), and (3) a combination of lacunes with severe WMH or the geographic patterns of cerebral microbleeds (mixed or strictly lobar) if cerebral microbleeds were present. Participants without cerebral microbleeds and severe WMH were classified as the control group. CSVD, cerebral small vessel disease; FLAIR, fluid-attenuated inversion-recovery; SWI, susceptibility-weighted images; TIV, total intracranial volume; WMH, white matter hyperintensities.

There is no effective treatment or method for the prevention of CSVD despite its acknowledged significance. It could be due to a lack of phenotyping methods that could stratify CSVD in response to its heterogeneity in clinical and neuroimaging manifestations and etiology^6^. Current CSVD indices are often derived by summing the different MRI markers that are present in an individual (CSVD score)^12^. These scoring methods may reflect the CSVD burden and predict prognosis^12^. However, they do not provide information about the underlying pathogenesis of CSVD. Recently, we developed a stratification scheme to classify a community-based, asymptomatic middle-to-old aged population into non-CSVD and four CSVD subtypes based on the following criteria in order: (1) bleeding or non-bleeding, (2) CMB locations, and (3) the severity and combination of WMH and lacune (Fig. 1b)^7^. Common and distinct patterns of the clinical and neuroimaging manifestations were found in the four stratified CSVD subgroups^7^. This MRI-based stratification scheme highlights the distinct features of SVD and the possible underlying pathogenesis. Stratified CSVD type 1, 2, and 3 might represent different severity of arteriosclerosis/lipohyalinosis CSVD, while CSVD type 4 suggests CAA^7^. However, whether this classification could differentiate longitudinal outcomes was not studied yet. This study aimed to investigate the 5-year mortality of each CSVD type in asymptomatic individuals stratified by our proposed CSVD classification scheme.

## Methods

### Study population (cohort description)

The I-Lan Longitudinal Aging Study (ILAS) is a community-based aging cohort study in I-Lan County, Taiwan, that aims to evaluate the mechanisms of aging^8^. Community-dwelling adults aged ≥ 50 years from Yuanshan Township in I-Lan County were invited to participate. The initial wave of participants was recruited between August 2011 and July 2014. The inclusion criteria of the ILAS were as follows: (1) inhabitants of I-Lan County who were not planning to move soon and (2) aged ≥ 50 years. In addition, participants who met any of the following conditions were excluded: (1) inability to communicate and complete an interview; (2) inability to complete a simple motor task (for example, a 6-m walk) due to functional disability, (3) presence of any major illness with associated decreased life expectancy (less than 6 months), (4) presence of any contraindication for MRI (such as metal implants), and (5) institutionalization for any reason. In addition, patients diagnosed with neuropsychiatric diseases, such as dementia, stroke, brain tumor, or major depression, were excluded from this study.

### Standard protocol approval, registration, and patient consent

The study was approved by the Institutional Review Board of the National Yang-Ming University, Taipei, Taiwan (IRB no. YM109161F). All participants provided written informed consent. All methods were carried out in accordance with relevant guidelines and regulations.

### Assessment of mortality

Between January 2018 and December 2019, ILAS investigators made phone calls inviting the initially recruited participants for follow-up clinical visits and brain MRI scans. Mortality status was collected from phone-call interviews. Since the cause of mortality might not be validated without available medical documentation, we did not analyze the different causes of mortality in this study.

### Brain MRI acquisition^7,15^

Multimodal neuroimaging acquisition was performed at the National Yang-Ming University to obtain CSVD markers for each participant, including WMH, lacunes, and CMBs. All MRI scans were collected on a single 3Tesla Siemens MRI scanner (Siemens Magnetom Tim Trio, Erlangen, Germany) with a vendor-supplied 12-channel phased-array head coil. All acquired whole-brain MRI scans were without inter-slice gap and interpolation. The following imaging sequences were used. First, T1-weighted images were acquired using a three-dimensional T1-weighted magnetisation-prepared rapid-acquisition gradient echo sequence (repetition time [TR]/echo time [TE]/inversion time [TI] = 3500/3.5/1100 ms; flip angle = 7°; number of excitations (NEX) = 1; field of view (FOV) = 256 × 256 mm; matrix size = 256 × 256; 192 sagittal slices; and voxel size = 1.0 mm^3^). Second, T2-weighted fluid-attenuated inversion recovery (FLAIR) images were acquired using a two-dimensional T2-weighted FLAIR multishot turbo-spin-echo sequence (TR/TE/TI = 9000/143/2500 ms; flip angle = 130°; NEX = 1; FOV = 220 × 220 mm; matrix size = 320 × 320, echo train length = 35; 63 axial slices; and voxel size = 0.69 mm × 0.69 mm × 2.0 mm). Third, susceptibility-weighted images (SWI) were acquired using a three-dimensional SWI sequence (TR/TE = 28/21 ms; flip angle = 15°, FOV = 256 × 224 mm; matrix size = 256 × 224; 88 axial slices; bandwidth = 120 Hz/Px; and voxel size = 1.0 × 1.0 × 2.0 mm). Before the image pre-processing, all the acquired MRI scans were visually examined by an experienced neuroradiologist to exclude any data with severe motion artifacts or gross brain abnormalities including trauma, tumor, and intracerebral hemorrhagic or territorial infarct lesions (in the territory of large arteries or their branches but not of a perforating artery).

### Volume quantification of WMH (Fig. 2)

**Figure 2:**
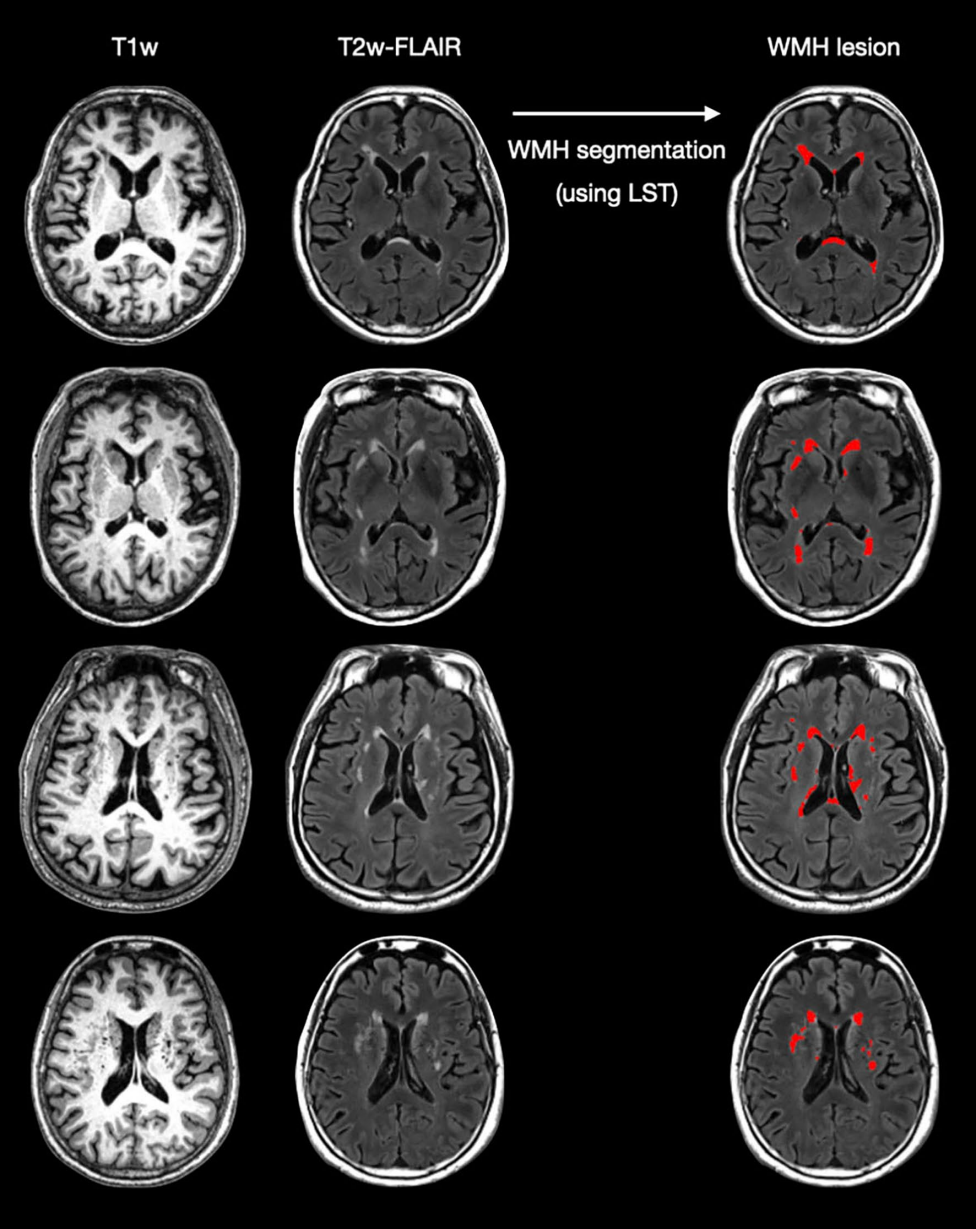
Example of white matter hyperintensity segmentation.

We applied the previously established analytical framework to estimate volumetric information of multiple tissue types for each individual^7,15^. All the following analyses were conducted with Statistical Parametric Mapping (SPM12, version 7487, Wellcome Institute of Neurology, University College London, UK, http://www.fil.ion.ucl.ac.uk/spm/) and Matlab R2016a (The Mathworks, Inc., Natick, MA, USA) using default settings. First, individual T2-weighted FLAIR scan was affine-registered to the corresponding T1-weighted scan, and then served as the inputs for generating a native T1 space WMH probability map and lesion-filled T1 images using the Lesion Segmentation Toolbox (LST, version 3.0.0, https://www.applied-statistics.de/lst.html)^24^. Second, all the lesion-filled T1 anatomical scans were processed using the standard Diffeomorphic Anatomical Registration Through Exponentiated Lie Algebra–voxel based morphometry (DARTEL-VBM) approach to obtain the corresponding deformation field for each individual^25^. Finally, all the native T1 space tissue probability maps (including gray matter, white matter, cerebrospinal fluid and WMH) were spatial transformed into the standard Montreal neurological institute (MNI) space using subject-specific deformation field and then modulated to obtain absolute volumetric information of total intracranial volume (TIV, summation of gray matter volume, white matter volume and cerebrospinal fluid volume) and WMH volume simultaneously.

### Detection and assessment of MRI SVD markers

CMBs were defined as small, rounded or circular, well-defined, hypointense lesions within the brain parenchyma with clear margins and ≤ 10 mm in size on SWI^9,10^. Microbleed mimics, such as vessels, calcification, partial volume, air-bone interfaces, and hemorrhages within or adjacent to an infarct, were carefully excluded. We used the microbleed anatomical rating scale to measure the presence, amount, and topographic distribution of CMBs^10^. Intra-rater reliability was assessed by evaluating CMBs in 20 randomly sampled images at a separate time (K, 0.83; 95% confidence interval [CI], 0.79–0.90). We also reassessed CMBs in the 25 randomly sampled images previously assessed by Dr. Chung and another investigator (K, 0.82; 95% CI, 0.79–0.88). CMBs were classified into deep, infratentorial, and lobar categories. Lobar topography was determined according to Stark and Bradley^11^ and included cortical and subcortical regions including subcortical U fibers. Lobar CMBs were assessed in the frontal, parietal, temporal, and occipital regions. Deep regions included the basal ganglia, thalamus, internal capsule, external capsule, corpus callosum, and deep/periventricular WM, while infratentorial regions included the brainstem and cerebellum. Individuals with CMBs were divided into two types according to the CMB topography: strictly lobar (CMB exclusively located in lobar regions) and mixed CMB (deep and/or infratentorial CMB with or without lobar CMB). Lacunes were assessed using T2-weighted FLAIR anatomical scans. Lacunes are defined as small (< 15 mm in diameter) cerebrospinal fluid-containing cavities, located in the deep gray or white matter, with adjacent WMH^2^.

### Determinations of CSVD types

The CSVD stratification scheme (CSVD types) had three steps in order: checking for the presence of (1) CMB, (2) severe WMH (defined as > 50th percentile of WMH/total intracranial volume ratio [0.07%]), and (3) a combination of lacunes with severe WMH or a certain geographic pattern of CMB (mixed or strictly lobar) when CMB is present (Fig. 1b). The differentiation between bleeding and non-bleeding SVD was achieved in the first step of the stratification scheme. We further stratified bleeding SVDs into different subtypes with CMBs of specific geographic features. Regarding the non-bleeding SVDs, subjects with severe WMHs were further divided depending on whether a lacune was present. Participants without CMB and severe WMH were allocated to the robust (control) group. There were two types of nonbleeding SVD (WMH without or with lacune; CSVD type 1 and 2) and two types of bleeding SVD (mixed or strictly lobar CMB; CSVD type 3 and 4).

### CSVD burden

We used a simple CSVD score to represent the CSVD burden^12,13^. One point was given for the presence of any lacune, severe WMH, and CMB; thus, the simple SVD score ranged from 0 to 3.

### Statistical methods

Analyses were performed using SPSS version 22.0. (IBM, Armonk, NY, USA). All data are presented as mean (standard deviation) for continuous variables and number (percentage) for discrete variables. Group comparisons were made using the nonparametric Kruskal–Wallis test with post-hoc analyses. When appropriate, chi-square or Fisher’s exact tests were performed for categorical variables.

The follow-up time for each individual was calculated from the date of initial recruitment until the date of the phone interview. The incidence rate of all-cause mortality was determined from the incidence per person year. A Kaplan–Meier survival curve was plotted, and the log-rank test was applied to test the difference in survival between groups. We then used the Cox regression analysis to calculate the crude and adjusted hazard ratios (HRs) and 95% CIs for the occurrence of all-cause mortality in each CSVD group compared to the control group. The covariates included the age, sex, and vascular risk factors (presence of hypertension, diabetes mellitus, and dyslipidemia and cigarette smoking). There was no significance in the HR changes with time in CSVD types (*p* = 0.061) and CSVD scores (*p* = 0.057), which showed that the assumption of proportionality was not violated. However, due to the borderline statistical-significance, we also put follow-up time as one of the covariates in regression analyses.

## Results

Among the initial sampling population of the ILAS recruited between August 2011 and July 2014, 760 individuals had received comprehensive MRI modalities for CSVD detection and evaluation. We excluded nine individuals with incidentally found brain tumors and 16 individuals with problematic images due to head motion. The flow chart of the study population is shown in Fig. 1a.

In 735 individuals with eligible brain MRI images, 335 (45.6%) were categorized into robust (control group) and 249 (33.9%), 52 (7.1%), 61 (8.3%), and 38 (5.2%) were classified into CSVD type 1, type 2, type 3, and type 4 groups, respectively (Table 1). The demographics and imaging characteristics of the five groups are shown in Table 1. The Kruskal–Wallis nonparametric analyses showed that age and the presence of hypertension were significantly different among the five groups. Post-hoc analyses showed that individuals in each CSVD group were older than those in the control group. Nevertheless, individuals in CSVD types 1, 2, and 3 groups (however, not in CSVD type 4) had a higher prevalence of hypertension than those in the control group.

**Table 1:** Comparisons between the control and four cerebral small vessel disease groups.

	Control	Non-bleeding CSVD		Bleeding CSVD		*p* value
		Isolated WMH	WMH with lacune	Mixed CMBs	SL CMBs	
		Type 1	Type 2	Type 3	Type 4	
Number	335 (45.6%)	249 (33.9%)	52 (7.1%)	61 (8.3%)	38 (5.2%)	
Age, years, mean (SD)	57.9 (5.9)	65.6 (8.1)	67.8 (9.2)	67.1 (10.3)	64.3 (9.0)	< 0.001^a^
Sex, men, n (%)	132 (39.4%)	120 (48.2%)	26 (50.0%)	31 (50.8%)	17 (44.7%)	0.241
Follow-up time, year	5.8 (0.8)	5.7 (0.6)	5.7 (0.8)	5.5 (0.7)	5.7 (0.6)	0.233
**Vascular risk factors**						
Hypertension, n (%)	77 (23.0%)	106 (42.6%)	28 (53.8%)	25 (41.0%)	9 (23.7%)	< 0.001
Diabetes, n (%)	22 (6.6%)	46 (18.5%)	14 (26.9%)	11 (18.0%)	8 (21.1%)	< 0.001
Dyslipidemia, n (%)	15 (4.5%)	14 (5.6%)	2 (2.8%)	7 (11.5%)	3 (7.9%)	0.241
Cigarette smoking, n (%)	67 (20.0%)	75 (30.2%)	18 (34.6%)	17 (27.9%)	9 (23.7%)	0.029
Systolic BP, mmHg, mean (SD)	124.3 (14.7)	132.0 (16.3)	134.7 (19.3)	132.6 (18.0)	129.2 (21.3)	< 0.001^b^
LDL, mg/dl, mean (SD)	120.1 (33.6)	117.3 (31.2)	114.0 (27.8)	113.6 (29.7)	112.5 (27.4)	0.319
HgbA1c, %, mean (SD)	5.8 (0.6)	6.0 (0.8)	6.6 (1.4)	6.1 (1.0)	6.0 (0.8)	< 0.001^c^
**CSVD MRI markers**						
Severe WMH, n (%)	0	249 (100%)	52 (100%)	49 (80.3%)	22 (57.9%)	< 0.001
WMH volume ratio, 10^−3^, mean (SD)	0.3 (0.2)	2.5 (3.1)	4.1 (4.4)	4.5 (4.8)	2.8 (3.9)	< 0.001^d^
Lacune, present, n (%)	0	0	52 (100%)	26 (42.6%)	5 (13.2%)	< 0.001
Lacune, n, mean (SD)	0	0	1.5 (0.9)	0.9 (1.2)	0.2 (0.5)	< 0.001^e^
CMB, present, n (%)	0	0	0	61 (100%)	38 (100%)	< 0.001
CMB, n, mean (SD)	0	0	0	2.7 (3.1)	1.2 (0.4)	< 0.001f.
CSVD score, mean (SD)	0	1 (0)	2 (0)	2.2 (0.7)	1.7 (0.7)	< 0.001^ g^
**CSVD score category, n (%)**						< 0.001
0	335 (100%)	0	0	0	0	
1	0	249 (100%)	0	10 (16.4%)	16(42.1%)	
2	0	0	52 (100%)	27 (44.3%)	18 (47.4%)	
3	0	0	0	24 (39.3%)	4 (10.5%)	

BP, blood pressure; CMB, cerebral microbleed; CSVD, cerebral small vessel disease; HgbA1c, hemoglobin A1c; LDL, low-density lipoprotein cholesterol; MRI, magnetic resonance imaging; SD, standard deviation; SL, strictly lobar; WMH, white matter hyperintensities.

^a^*Post-hoc* analyses showed that all CSVD types were significantly older than the control group.

^b^*Post-hoc* analyses showed that CSVD type 1, 2 and 3, respectively, were significantly higher than the control group.

^c^*Post-hoc* analyses showed that CSVD type 3 was significantly higher than all the other groups and CSVD type 2 was significantly higher than the control group.

^*d*^*Post-hoc* analyses showed that all CSVD types were significantly higher than the robust group; CSVD type 2 and 3 were both significantly higher than CSVD type 1; CSVD type 3 was also significantly higher than CSVD type 4.

^e^*Post-hoc* analyses showed that CSVD type 2 and 3 were significantly more than the robust group.

^f^*Post-hoc* analyses showed that CSVD type 3 and 4 were significantly more than the robust group.

^g^*Post-hoc* analyses showed that all groups were significantly different compared with each other.

^h^Log-rank test.

Additional characteristics of the CSVD markers in each classified subtype are presented in Table 1. In individuals with bleeding SVD, particularly type 3, the presence of severe WMH was also prominent (80.3% and 57.9% in types 3 and 4, respectively). The WMH volume ratios were significantly higher in each SVD group than in the control group. Lacunes were also present in bleeding SVD; 42.6% and 13.2% of types 3 and 4 had at least one lacune. The mean CSVD score and the distribution of the CSVD score category in each group are shown in Table 1. CSVD burden was different among the five groups; CSVD type 3 had the highest CSVD scores (Table 1). Notably, there was no participant with isolated lacune (with lacune but no severe WMH or CMBs).

### Mortality and CSVD types

**Figure 3:**
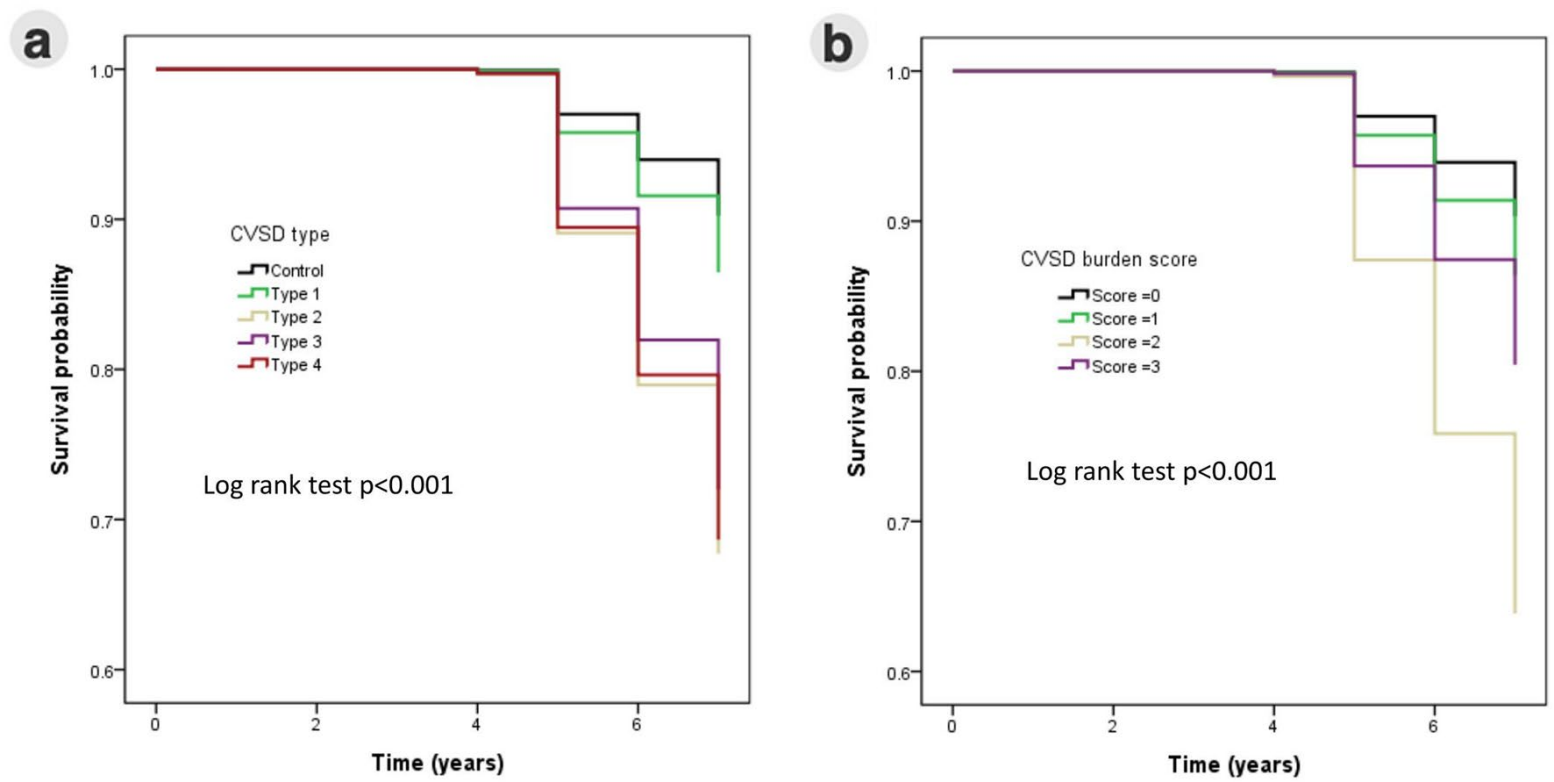
Kaplan–Meier survival curves of each category of cerebral small vessel disease. (**a**) Four types of cerebral small vessel disease (CSVD). (**b**) The burden of CSVD is defined by the CSVD score of 0–3.

We contacted all recruited participants by phone call and recorded their all-cause mortality status. The follow-up time did not differ between the groups (Table 1). During a mean follow-up of 5.7 (0.7) years, 62 (8.4%) died. The survival curves for each group are shown in Fig. 3. Table 2 demonstrates the incidence and HR of all-cause mortality in each group and the group comparisons. The results showed that after age and sex adjustment, only the CSVD type 4 group had a significantly higher all-cause mortality rate than the control group (HR 4.1, 95% confidence interval 1.4–12.1, *p* = 0.011), and the significance remained after adjustment for vascular risk factors (Table 2; HR 5.0, 95% confidence interval 1.6–15.3, *p* = 0.005). Since the ages were different between controls and CSVD groups, we also selected participants in the control group with age-matched with CSVD groups for the regression analyses (Table 2). The results showed that higher mortality in CVSD type 4 remained and even more significant compared with age-matched controls (Table 2; HR 6.8, 95% confidence interval 1.7–28.1, *p* = 0.008).

**Table 2:** Rates and adjusted hazard ratio of all-cause mortality in each type and burden category of cerebral small vessel disease.

	Event number	Person-year	Rate (% per person year)	HR^a^ (95% CI)	*p* value	HR^b^ (95% CI)	*p* value
**CSVD types**							
Control	18	1925	0.9	1.0	–	1.0	–
Type 1	18	914	2.0	1.0 (0.4–2.3)	0.985	1.0 (0.4–2.4)	0.985
Type 2	10	294	3.4	2.6 (0.9–7.7)	0.077	2.4 (0.8–7.8)	0.128
Type 3	9	337	2.7	1.7 (0.5–5.3)	0.367	1.6 (0.5–5.2)	0.471
Type 4	7	215	3.3	4.1 (1.4–12.1)	0.011	5.0 (1.6–15.3)	0.005
**CSVD types**							
Age-matched control	–	–	–	1.0	–	1.0	–
Type 1	–	–	–	1.5 (0.5–4.7)	0.493	1.6 (0.5–5.2)	0.442
Type 2	–	–	–	4.0 (1.1–15.2)	0.039	3.7 (0.9–15.1)	0.073
Type 3	–	–	–	2.4 (0.6–9.5)	0.196	2.0 (0.5–8.6)	0.345
Type 4	–	–	–	5.5 (1.5–20.9)	0.012	6.8 (1.7–28.1)	0.008
**CSVD burden**							
Score 0	18	1925	0.9	1.0	–	1.0	–
Score 1	20	1559	1.3	1.1 (0.5–2.3)	0.833	1.0 (0.5–2.2)	0.985
Score 2	21	544	3.9	3.0 (1.3–6.8)	0.009	2.7 (1.1–6.2)	0.024
Score 3	3	157	1.9	1.0 (0.2–4.3)	0.995	1.1 (0.3–4.7)	0.923

CI, confidence interval; CSVD, cerebral small vessel disease; HR, hazard ratio.

^a^Adjusted for follow-up time, age and sex.

^b^Adjusted for follow-up time, age, sex, and vascular risk factors (presence of hypertension, diabetes mellitus, and dyslipidemia and cigarette smoking).

### Mortality and CSVD burden

We also analyzed the associations between the all-cause mortality rate and CSVD burden using CSVD scores. There were 335, 275, 97, and 28 individuals with CSVD scores of 0, 1, 2, and 3, respectively. The results of Cox regression analyses did not show a dose-dependent relationship between the CSVD score and all-cause mortality, e.g. higher CSVD scores did not have a higher all-cause mortality rate (Table 2). In addition, after age and sex adjustment, only individuals with CSVD score 2 showed a significantly higher all-cause mortality rate compared to the those with CSVD score 0. In contrast, the CSVD score 1 and 3 groups had similar all-cause mortality rates as the CSVD score 0 group (Table 2).

### Subgroup analysis of CSVD type 4

Within CSVD type 4, the associations between each demographic/neuroimaging characteristic and all-cause mortality are shown in Table 3. We did not find any factors associated with the all-cause mortality rate in the CSVD type 4 group. However, a higher number of CMBs (CMB ≥ 2) had a trend showing higher rate of mortality though statistically non-significant. Again, the CSVD burden was not associated with all-cause mortality in this subgroup.

**Table 3:** Associations between each demographic and neuroimaging factor and all-cause mortality in cerebral small vessel disease type 4.

	HR (95% CI)	*p* value
Age > 63 year-old^a^	1.7 (0.4–7.8)	0.476
Sex, female	1.0 (0.2–4.6)	0.976
Hypertension	1.0 (0.2–5.4)	0.959
Diabetes Mellitus	0.6 (0.1–5.1)	0.640
Hyperlipidemia	1.2 (0.1–11.2)	0.850
Cigarette smoking	1.6 (0.9–3.1)	0.126
The presence of severe WMH (n = 22)	1.4 (0.3–7.8)	0.686
The presence of lacune (n = 5)	0.04 (0–2911.6)	0.576
The number of CMB ≥ 2 (n = 5)	4.0 (0.7–22.2)	0.109
**CSVD burden (versus CSVD score 1)**		
CSVD score 2 (n = 18)	1.7 (0.3–9.3)	0.537
CSVD score 3 (n = 4)	0.04 (0–142,489.2)	0.668

CI, confidence interval; CSVD, cerebral small vessel disease; CMB, cerebral microbleed; HR, hazard ratio; WMH, white matter hyperintensities.

^a^The median age of the CSVD type 4 group.

## Discussion

This study evaluated the association between CSVD and 5-year mortality in an asymptomatic (stroke-free and non-demented) middle-to-old aged population with two MRI marker-based classification methods, CSVD phenotypes, and burden. The results showed that among all CSVD types, the CSVD type 4 group was significantly associated with a higher rate of all-cause mortality independent of age, sex, and vascular risk factors. However, the CSVD burden measured by the simple CSVD score did not show a positive association with all-cause mortality. Only CSVD scores of 2, but not scores 1 and 3, were significantly and independently associated with a higher rate of all-cause mortality.

Our CSVD stratification scheme used three common MRI markers (Fig. 1b)^7^. The first step of the stratification scheme differentiated CSVD into bleeding and nonbleeding subtypes. Bleeding CSVDs were further stratified into different subtypes with CMBs of specific topographic features that have distinct underlying microvasculopathy: strictly lobar CMBs considering as CAA and mixed CMBs as arteriosclerosis/lipohyalinosis microvasculopathy^14,15^. Regarding nonbleeding CSVDs, individuals with severe WMHs were further divided depending on whether a lacune was present. This phenotyping method overcomes the limitations of other classification methods in that it only counts the number of CSVD markers but does not consider their nature. This classification method also corresponds to the authentic situation in which individuals with CSVD usually have different combined CSVD MRI markers. Our previous study has validated this CSVD phenotyping method by showing distinct clinical features and neuroanatomic changes in the stratified CSVD types in the asymptomatic (stroke-free and non-demented) middle-to-old aged population; CSVD types 1, 2, and 3 were more likely originated from arteriosclerosis/lipohyalinosis CSVD, while CSVD type 4 was suggestive of CAA^7^. This study showed that the association between CSVD and 5-year all-cause mortality in the same population was also mediated by the CSVD types. In the CSVD type 4, the presentation of possible CAA as the underlying CSVD, at a mean age of 64.3 years, predicted a 4–fivefold higher all-cause mortality than in the non-CSVD group. Notably, these individuals lacked hemorrhagic stroke, the typical diagnostic criteria of CAA^16^. These results indicate that this CSVD phenotyping method is able to differentiate survival outcomes in different underlying microvasculopathies, in an asymptomatic early stage of disease.

Previous studies have studied the mortality rate in patients with CMBs of different topographic patterns^17,18^. They showed that mixed CMBs were associated with cardiovascular mortality, while lobar CMBs were associated with stroke-specific mortality compared to patients without CMBs^17,18^; these results correspond with the notion that mixed CMBs are considered to reflect hypertensive arteriopathy and, therefore, systemic vascular disease, while lobar CMBs are indicative of CAA and primarily restricted to the brain^14^. Although not provided in the literature, the strokes related to lobar CMB-associated mortality were probably intracerebral hemorrhage, a major clinical consequence of CAA^19^. Therefore, we postulated that causes of mortality in the CSVD type 4 in this study were primarily hemorrhagic strokes. In the CSVD type 3 group, patients with mixed CMBs, had higher mortality than the control group (14.8% vs. 5.4%; Table 1), and the significance diminished after adjustment for age, sex, and vascular risk factors (Table 2). Our population had low cardiovascular risk or otherwise with optimal medical control (Table 1), therefore, it might reduce the expected higher cardiovascular mortality in patients with CSVD type 3 in this study.

The initial studies of CSVD regarding their clinical outcome usually focused on only one MRI marker, particularly WMH or CMBs^17,18,20^. However, individuals with CSVD usually have not only one but several coexisting MRI markers. Attempting to capture the overall effect of CSVD on the brain (CSVD burden), researchers have proposed the CSVD score, which is yielded by summing up the number of simultaneous MRI markers’ appearance in a person^12,13^. The original CSVD score was computed by counting the presence of severe WMH, lacune, CMB, and dilated perivascular space (PVS) as an ordinal score of 0–4^12^. Since PVS data are not routinely assessed as commonly as the other MRI markers in clinical or research settings, measurement without PVS is also generated as the simple CSVD score^13^. Two previous large studies evaluated the association between CSVD score and mortality; one was in patients with acute ischemic stroke^21^, and another was in a stroke-free and non-demented community-based population^22^. In patients with acute ischemic stroke, only patients with the highest CSVD score (score 4) but not score 1, 2, or 3 had a significantly higher rate of all-cause mortality than those with CSVD score 0^21^. In another study with an asymptomatic population, similar to the present study, only a CSVD score of 2 showed significant association with all-cause mortality but not the other CSVD scores^22^. The lack of significant association between a higher CSVD score and all-cause mortality in an asymptomatic population might be explained by a low prevalence of mortality rate, small number of patients with a high CSVD score, or both. Therefore, it is suggested that the CSVD score might not be feasible to predict survival outcomes in asymptomatic or preclinical stages of CSVD. Notably, in the subgroup analyses of CSVD type 4, a higher CSVD score was also not correlated with a higher all-cause mortality. Among the three MRI markers, only the number of CMBs showed a positive association with all-cause mortality in the CSVD type 4 group (Table 3). These results again indicate that the nature (bleeding or nonbleeding; arteriosclerosis or CAA) might be more important than the number of CSVD markers when considering the clinical significance of CSVD.

There were limitations to the present study. First, we did not obtain information regarding the cause of death. Second, the present findings were from a community-based community that had no history of stroke or dementia. Whether the results could be generalized to other populations with evident clinical events requires further validation. Third, we did not assess and thus not include PVS in the evaluation of CSVD. Finally, the presence of severe WMH was defined as > 50th percentile of WMH/total intracranial volume ratio in our study population. We used automatic volumetric measurement to scale WMH since it offers a reliable and objective alternative to visual rating scales^23^. The cut-off points of the WMH volume ratio for defining severe WMH in different populations might be different.

In conclusion, we showed that the MRI-based CSVD classification scheme could evaluate the 5-year all-cause mortality in stratified CSVD types in stroke-free and non-demented populations. This CSVD stratification method, which could identify asymptomatic individuals at risk of mortality, may be of clinical diagnostic value. The results showed that patients with CSVD type 4, possible CAA, had a higher all-cause mortality and provided insight into the early disease stage of CAA.

## Data Availability

Clinical and neuroimaging raw data are available from the corresponding author on request.
